# Expression of TILs and Patterns of Gene Expression from Paired Samples of Malignant Pleural Mesothelioma (MPM) Patients

**DOI:** 10.3390/cancers15143611

**Published:** 2023-07-14

**Authors:** Susana Cedres, Garazi Serna, Alberto Gonzalez-Medina, Augusto Valdivia, Juan David Assaf-Pastrana, Patricia Iranzo, Ana Callejo, Nuria Pardo, Alejandro Navarro, Alex Martinez-Marti, Ilaria Priano, Roberta Fasani, Xavier Guardia, Javier Gonzalo, Caterina Carbonell, Joan Frigola, Ramon Amat, Victor Navarro, Rodrigo Dienstmann, Ana Vivancos, Paolo Nuciforo, Enriqueta Felip

**Affiliations:** 1 Vall d’Hebron Institute of Oncology (VHIO), Hospital Universitari Vall d’Hebron, 08035 Barcelona, Spain; 2 Molecular Oncology Group, Vall d’Hebron Institute of Oncology (VHIO), 08035 Barcelona, Spain; 3 Cancer Genomics Lab, Vall d’Hebron Institute of Oncology (VHIO), 08035 Barcelona, Spain; 4 Vall d’Hebron Institute of Oncology (VHIO), 08035 Barcelona, Spain; 5 Clinical Research Department, Vall d’Hebron Institute of Oncology (VHIO), Passeig Vall d’Hebron 119-129, 08035 Barcelona, Spain; 6 Oncology Data Science Group, Vall d’Hebron Institute of Oncology (VHIO), 08035 Barcelona, Spain

**Keywords:** malignant pleural mesothelioma, immunotherapy, gene expression, TIL

## Abstract

**Simple Summary:**

Recently immunotherapy has been approved in first line for patients with MPM. However, the benefit of immunotherapy in MPM is modest, and recognizing the immune landscape of this tumor could guide the optimal therapeutic strategy. In some tumors dynamic changes of TILs has been reported after chemotherapy, endocrine therapy and immunotherapy. We investigated the changes in expression of TILs in human MPM tumor tissue using immunohistochemistry and patterns of gene expression from paired samples. We included samples of patients treated with chemotherapy, immunotherapy and patients without any treatment between the samples. In our series, after systemic treatment or at progressive disease we observed a decrease of the number of TILs and a downregulation of the immune-related genes. In patients without any treatment between the samples we found an increase of immune cells. We suggest that the immune system tends to turn off during the evolution of disease of after treatment.

**Abstract:**

MPM is an aggressive disease with an immunosuppressive tumor microenvironment, and interest in exploring immunotherapy in this disease has been increasing. In the first line of treatment, the combination of nivolumab and ipilimumab demonstrated an improvement in survival over chemotherapy. The presence of TILs has been recognized as a marker of antitumor immune response to chemotherapy in solid tumors. The aim of our study is to identify the effect of treatment on immune cells and the immune gene profile in MPM. We investigated the changes in expression of TILs in 10 human MPM paired tumor tissues using immunohistochemistry and gene expression analysis from paired untreated and treated samples. In this small series, we demonstrated that during the evolution of disease without any treatment there was an increase in the inflammatory component in tumor samples. After systemic treatment there was a decrease in the number of TILs. We observed that after systemic treatment or disease progression immune gene signatures were suppressed. Our integrated analysis of paired samples with immune profile and genomic changes on MPM suggested that during the evolution of the disease the immune system tends to switch, turning off with treatment.

## 1. Introduction

Malignant pleural mesothelioma is an aggressive and fatal cancer related to asbestos exposure, with a long latency between the exposure and the development of the disease. Incidence has been increasing in recent years, and this trend is expected to continue in some countries over next few decades [1,2].

The standard treatment for patients with advanced disease is chemotherapy based on platinum plus antifolate, which demonstrated a benefit to median survival of 12.1 months [3]. The addition of bevacizumab also demonstrated a benefit to median survival of up to 18.8 months, and although bevacizumab has not received approval, it has been incorporated into clinical practice guidelines [4]. The chronic inflammatory response causes an immunosuppressive tumor microenvironment, which has led to a significant interest in exploring immunotherapy in MPM [5,6]. The CheckMate 743 study demonstrated a significant improvement in overall survival (OS) for the combination of nivolumab plus ipilimumab over chemotherapy in the first line of treatment, leading to approval by regulatory agencies [7]. In the analysis of biomarkers, the four-gene inflammatory signature (*CD8A*, *STAT1*, *LAG3*, and *CD274*) appeared to correlate with improved survival [8].

The development of targeted therapies in MPM has been disappointing, mainly due to the paucity of actionable targets. The mutational landscape of MPM described low TMB and identified tumor suppressors *BAP1*, *NF2*, *TP53*, *LATS2*, and *SETD2* as significant mutated genes [9]. A transcriptomic analysis generated different classifications partially related to histology and with a different prognosis [10,11]. Recently, the evolutionary analysis of the phylogenetic tree of MPM suggested that *BAP1* events occur early in the evolution of MPM and *NF2* events occur late [12].

Tumor-infiltrating lymphocytes (TILs) represent an immune cell population that recognizes tumor antigen but may have developed an exhausted phenotype due to the tumor microenvironment (TME). In MPM, asbestos-induced damage generates a tumor microenvironment with relatively low numbers of TILs, resulting in a reduction in tumor immunity and a strong immunosuppressive microenvironment [13,14].

Emerging data suggest that TILs are associated with prognosis and response to both cytotoxic treatments and immunotherapy in solid tumors. Considering the evolution of TILs during the progression of disease, changes in TILs have been observed during cancer evolution in pancreatic cancer, with the proportional distribution of CD3+ T cells, CD4+ T cells, and PD-L1 in tumor being significantly higher than in precancerous tissues, whereas the proportional distribution of CD8+ T-cells was significantly lower [15]. In addition, several studies suggested that TIL density is a strong prognostic marker for survival [16,17,18,19]. In MPM, a study analyzing TILs in patients who underwent surgery confirmed that CD8+ is a prognosis marker, but its prognosis remains unclear in chemotherapy-treated patients [20,21,22,23,24].

Regarding the predictive role of TILs with treatment, dynamic changes in TILs have been observed after systemic treatment. In breast cancer, a significant increase in the CD8+/Treg ratio was observed in response to neoadjuvant steroidal aromatase inhibitor [25]. In addition, in patients with breast cancer treated with HER2 blockade, an increase in CD3+, CD4+, CD8+, and FOXP3+ was observed after 2 weeks of therapy, with a subsequent decrease after surgery in patients achieving pathological complete response [26]. Regarding treatment with chemotherapy, it has been demonstrated that chemotherapy influences the TME, with expression of immune checkpoints and upregulation of PD-L1 after chemotherapy in some tumors [27,28,29,30]. In MPM, two studies analyzed changes in immune cells with chemotherapy. Pasello et al. analyzed 14 paired samples of patients with MPM treated with neoadjuvant chemotherapy, and they demonstrated that after chemotherapy tumors showed an increase in CD8+, CD3+, and CD68+ cells [31]. In a second study evaluating the activity of lurbinectedin in previously treated patients, the biomarker analysis in a cohort of seven patients demonstrated that Tregs and M2 in tumors were predictive biomarkers for lurbinectedin [32].

Finally, the evolution of TILs has also been analyzed in patients under immunotherapy, and TILs have been recognized as a marker of antitumor immune response across a wide range of tumors. In melanoma, early changes in T-cell repertories predicted response to immunotherapy, and in breast cancer, CD8+ TILs in peripheral blood correlated with subsequent responses to immunotherapy [33,34,35]. However, there is a lack of information about TIL evolution in MPM patients treated with immunotherapy, and no changes in PD-L1 expression following immunotherapy have been observed [36]

Identification of the effect of treatment on TILs in MPM can guide the rational design of combination strategies of immunotherapy with chemotherapy. We investigated the changes in expression of TILs in human MPM tumor tissue using immunohistochemistry and patterns of gene expression from paired untreated and treated samples. The association between TILs and outcomes was evaluated as a secondary endpoint. This could help to understand the immune response and evaluate its prognostic and predictive values.

## 2. Methods

### 2.1. Patients

The study cohort included patients with confirmed MPM who had two paired tumor samples of their disease, had a clinical record available, and were 18 years or older. Ten cases with enough archival tumor tissue for the analysis were collected. Clinicopathologic information gathered included complete history, age, sex, performance status, asbestos exposure, tumor stage, and histological subtype. The tumor stage was defined according to the International Union Against Cancer’s tumor–node–metastasis 8th classification and sub-classified histologically according to the WHO guidelines. All cases were reviewed by local pathologists with expertise in the diagnosis of MPM. We evaluated two tumor biopsies for each patient. All tumor biopsies were obtained by surgery (17 samples) or core needle biopsy (3 samples), and local pathologists confirmed the adequation of the sample to provide a diagnosis of MPM, carry out a histological subclassification, and perform the needle immunohistochemical staining. This study was approved by the local ethical committee (Ethical Committee at Vall d’Hebron Hospital Universitari). All methods were performed in accordance with the relevant guidelines and regulations.

### 2.2. Study Outcomes

The primary objective of this study was to describe the pattern of TILs and genomic alterations in paired samples in MPM over time or after treatment. Secondary analysis included assessment of the outcomes and prognosis.

### 2.3. Statistical Analysis

In this study, boxplots were employed to visualize the distribution and variability of treatment changes between paired samples. To assess the statistical significance of these differences, a Wilcoxon matched-pairs signed-rank test was performed, allowing for a robust comparison between paired observations within the treatment groups. Data were censored at last follow-up for patients without relapse or death. Median follow-up time was calculated with a reverse Kaplan–Meier estimator. OS was calculated from diagnosis of malignancy until death due to any cause or until the date of the last follow-up visit for living patients. Survival analysis that compared efficacy of treatment by TILs was carried out using the Kaplan–Meier curves, and the significance was verified by a log-rank test. All *p*-values were determined by two-sided tests and *p*-values < 0.05 were considered significant.

### 2.4. Tumor Tissue

All samples were reviewed and classified according to the current WHO classification. After revision of the tumor sample by two pathologists, two hematoxylin eosin slides and three slides were sent for RNA and IHC analysis. Sections 3 µm thick were prepared from formalin-fixed paraffin-embedded (FFPE) tissue blocks. Prior to staining the sections were backed in an oven for an hour at 60 °C.

Singleplex IHC was performed for PD-L1 and CD20 in the Benchmark Ultra platform from Ventana. PD-L1 was manually evaluated as CPS, and CD20 was analyzed by image analysis and the CD20 density was extracted. The antibodies and protocols used in this study are described in Table 1.

Tumor samples were subjected to analysis using next-generation IHC (NGI) with an immune panel to assess key immune effector cells, including total lymphocytes (CD3), cytotoxic T cells (CD8), regulatory T cells (FOXP3), and monocytes/macrophages (CD163). In brief, NGI involves sequential immunohistochemical staining on the same tissue section. This is achieved by destaining the alcohol-soluble chromogen between the stainings, followed by digitalization and alignment of the images. Ultimately, image analysis allows for the extraction of information regarding various biomarkers within the same cells. This methodology has been validated and employed for various panels [26,37,38]. Lymphocyte expression was evaluated through image analysis, with density quantified as the number of cells per square millimeter. The antibodies and protocols utilized in this study are detailed in Table 2 based on the staining order. Positive controls were incorporated on each slide.

### 2.5. DNA Sequencing of FFPE Tumor Samples

All samples were FFPE, and blocks selected and evaluated for tumor area by a pathologist were used for analysis. DNA was automatically extracted from 5 × 10 µm sliced sections of FFPE material using the Maxwell FFPE Tissue LEV DNA Purification Kit (Promega, Madison, Wisconsin, WI, USA) according to the manufacturer’s instructions. Quantification was performed with a Qubit dsDNA broad-range assay kit and a Qubit 4.0 fluorometer (Life Technologies, Carlsbad, CA, USA). Libraries were prepared according to the SureSelect XT standard protocol (Agilent Technologies, Inc., Santa Clara, CA, USA). A total of 500 ng of extracted DNA was processed for library preparation using a custom hybridization-based capture panel targeting 435 genes with reported somatic mutations in different tumor types (VHIO-300 v4 panel, Supplementary Table S1). Indexed libraries were quantified by qPCR using the KAPA Library Quantification Kit (Roche Sequencing Solutions), pooled, and sequenced in a HiSeq2500 platform (Illumina, Inc.) to generate 2 × 100 bp at an average coverage of 500×.

Reads were aligned (BWA v0.7.17, Samtools v1.9) against the hg19 reference genome, base recalibrated, indel realigned (GATK v3.7.0), and variant called (VarScan2 v2.4.3). Final average coverage depth per gene was ×500 (ranging from ×300 to ×500). A minimum of 7 reads supporting the variant allele was required to call a mutation. Frequent single nucleotide polymorphisms (SNPs) in the population were filtered based on the gnomAD database (allele frequency ≤ 0.0001), and copy number alterations (CNA) were calculated (CNVkit v0.9.6.dev0). Clinical significance classification of the variants was performed using the following databases: COSMIC (https://cancer.sanger.ac.uk/cosmic, accessed on 14 March 2023), cBioPortal (http://www.cbioportal.org/, accessed on 14 March 2023), ClinVar (https://www.ncbi.nlm.nih.gov/clinvar/, accessed on 14 March 2023), OncoKB (https://www.oncokb.org/, accessed on 14 March 2023), and VarSome (https://varsome.com/, accessed on 14 March 2023). Finally, manual curation of the data was performed among all the knowledge databases for harmonization of the criteria.

### 2.6. RNA Library Preparation from FFPE Tumor Samples

RNA was automatically extracted from FFPE tumors using the Maxwell RSC RNA FFPE Kit (Promega Maxwell RSC). RNA quantification was performed with RNA ScreenTape analysis (Agilent Technologies, Inc.). A total of 200 ng of starting material was converted to double-strand cDNA using the cDNA Synthesis Kit (Illumina Inc., San Diego, CA, USA). Library prep was performed from cDNA with the RNA Prep Kit (Illumina, Inc.) and dual index adapters (IDT for Illumina UMI RNA UD Indexes, Illumina, Inc.). The libraries were enriched with an exome panel (Illumina, Inc.) using the Fast Hybridization Kit (Illumina, Inc.). The enriched libraries were quantified with the High Sensitivity D1000 ScreenTape System (Agilent Technologies, Inc.), pooled, and sequenced with the NextSeq 550 or HiSeq2500 platforms (Illumina, Inc.) to generate 2 × 75 bp reads with a minimum of 20 M of reads (paired end). Quality control analysis for the FASTQ reads was performed using FastQC v0.11.8. Read trimming of low-quality bases was completed using Trimmomatic v0.39. The STARv2.7.9a algorithm was used for reading the alignment, employing the human GRCh38.p13 genome assembly and transcriptome as references. The resulting BAM files were analyzed with QualiMap v2.2.1 to inspect for alignment quality metrics. Transcript read counts were obtained with HTSeq v0.13.5 and then were used for differential gene expression analysis using DESeq2 v1.36.0. Finally, gene set enrichment analysis for KEGG pathways and GO terms was conducted using fgsea v1.20.0 to compare enriched pathways between paired samples. All steps from the workflow were executed and orchestrated using the bioinformatics BatchX, Inc. (www.batchx.io, accessed on 14 March 2023) cloud platform.

## 3. Results

### 3.1. Patient Population

We studied 10 patients with MPM with matched formalin-fixed paraffin-embedded tissue samples. The median age was 62 years (range 53–84). Patients were predominantly male (70%), were smokers (80%), had previous asbestos exposure (90%), and were in stage III (60%). The total cohort comprised nine epithelioid tumors and one biphasic tumor. Out of the entire group, none of the patients were considered for extrapleural pneumonectomy, and seven patients were treated with chemotherapy (Supplementary Table S2). Median survival of the entire group was 25.1 months (16.9 m-NR).

### 3.2. TIL

We examined 20 matched formalin-fixed paraffin-embedded tissue samples from 10 patients with MPM taken at diagnosis and at disease progression (follow-up), including untreated and treated with chemotherapy or immunotherapy. All patients, including untreated patients, presented disease progression at the time of the second biopsy. The median interval between paired biopsies was 12 months (3–33 m). Three matched samples belonged to patients who did not receive any systemic treatment between biopsies, and the other seven pairs of samples belonged to patients treated with chemotherapy plus/minus immunotherapy. The reason for not indicating treatment between biopsies for three patients was because of surgical resection in early stages with comorbidity not suitable to adjuvant treatment (2 patients) and because of poor performance status (1 patient). The second biopsy of the three patients that did not receive any systemic treatment was performed during surgery for talc pleurodesis. The second biopsy of patients who received systemic treatment was obtained at the progressive disease. All patients signed an informed consent form before obtaining the second biopsy. A summary of the history of each patient is presented in Table 3.

**Figure 1:**
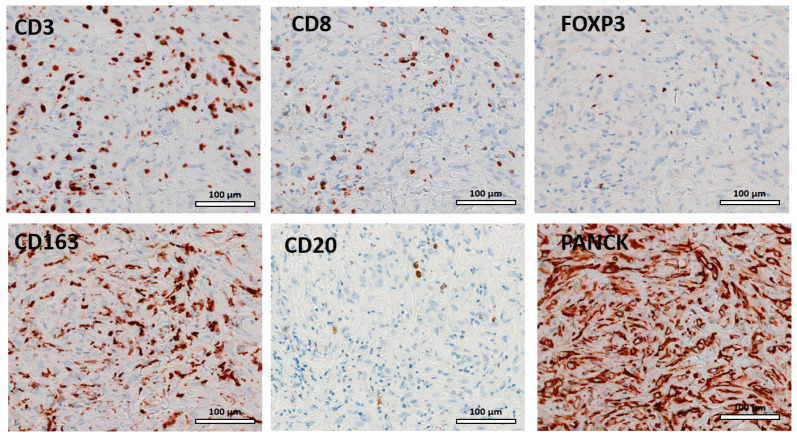
Representative examples of hematoxylin eosin immunohistochemical staining of TILs.

Tissue sections were stained for six different immune cell markers (Table 2, Figure 1). The tissue sections were analyzed for the presence of lymphocytes, and lymphocytic infiltrations was found in all tissue samples. The predominant cell type of the immune infiltrate was CD163 both in the untreated sample and in the second sample.

**Figure 2:**
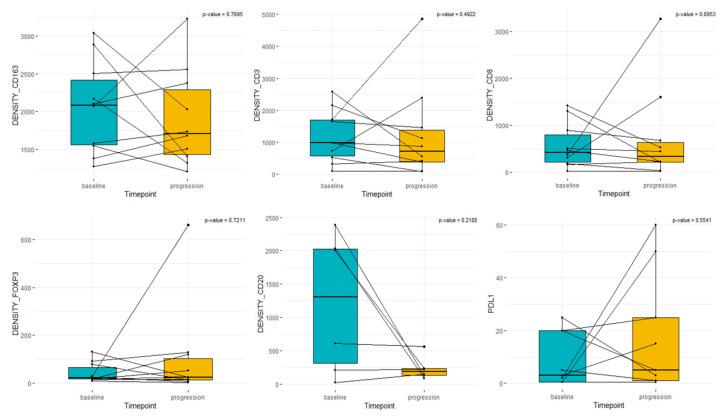
Box plot analysis of immune cells and PD-L1 in paired samples. “Baseline” represents sample from diagnosis. “Progression” represents the matched sample with or without previous treatment.

Samples from untreated patients showed more infiltration than from treated ones. Median densities of T cells and macrophages in diagnostic and matched follow-up samples were CD3+: 982 vs. 712, CD8+: 422 vs. 334, FOXP3+: 22 vs. 25, CD163+: 2086 vs. 1706, and CD20+ 864 vs. 199 (Figure 2).

**Figure 3:**
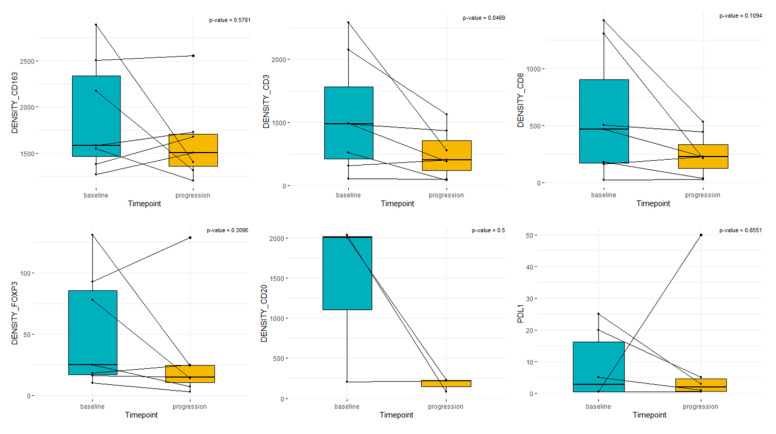
Box plot analysis of immune cells and PD-L1 TILs in paired samples treated with systemic treatment. “Baseline” represents sample from diagnosis. “Progression” represents the matched sample after treatment with chemotherapy plus/minus immunotherapy.

In patients receiving treatment between sample pairs, the median densities in diagnostic and follow-up matched samples were CD3+: 979 vs. 407, CD8+: 467 vs. 225, FOXP3+: 25 vs. 15, CD163+: 1583 vs. 1508, CD20: 793 vs. 141, and PD-L1: 2 vs. 1 (Figure 3).

**Figure 4:**
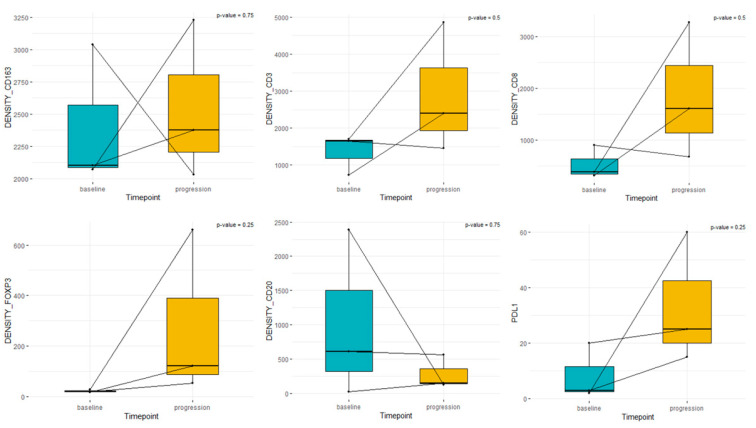
Box plot analysis of immune cells and PD-L1 in paired samples treated without systemic treatment among the samples. “Baseline” represents sample from diagnosis. “Progression” represents the matched sample without any systemic treatment at the moment of progressive disease.

In patients without any systemic treatment between sample pairs, the median densities were CD3+: 1645 vs. 2397, CD8+: 376 vs. 1605, FOXP3+: 19 vs. 121, CD163+: 2102 vs. 2377, CD20+: 1007 vs. 277, and PD-L1: 3 vs. 25 (Figure 4)

### 3.3. PD-L1

A cytoplasmic or membrane staining of PD-L1 was observed in seven untreated samples (corresponding to samples from diagnosis) and eight pretreated samples (corresponding to the tumor biopsy performed after at least one line of systemic treatment). Median PD-L1 CPS was 2.5 and 4 in diagnostic and follow-up samples, respectively. In general, no changes in PD-L1 were observed between matched samples except for two patients (Figure 2, Figure 3 and Figure 4). The first patient presented basal PD-L1 < 1%, and in the second sample after treatment with platinum pemetrexed in the first line and vinorelbine in the second line, PD-L1 increased to >50%. The second patient with meaningful changes in PD-L1 expression presented a low basal expression (2%), and in the second sample, one month later without any treatment, the PD-L1 expression was 60%. The heterogeneity of PD-L1 expression among the localization of tumor biopsy could explain these discrepancies.

### 3.4. Survival

**Figure 5:**
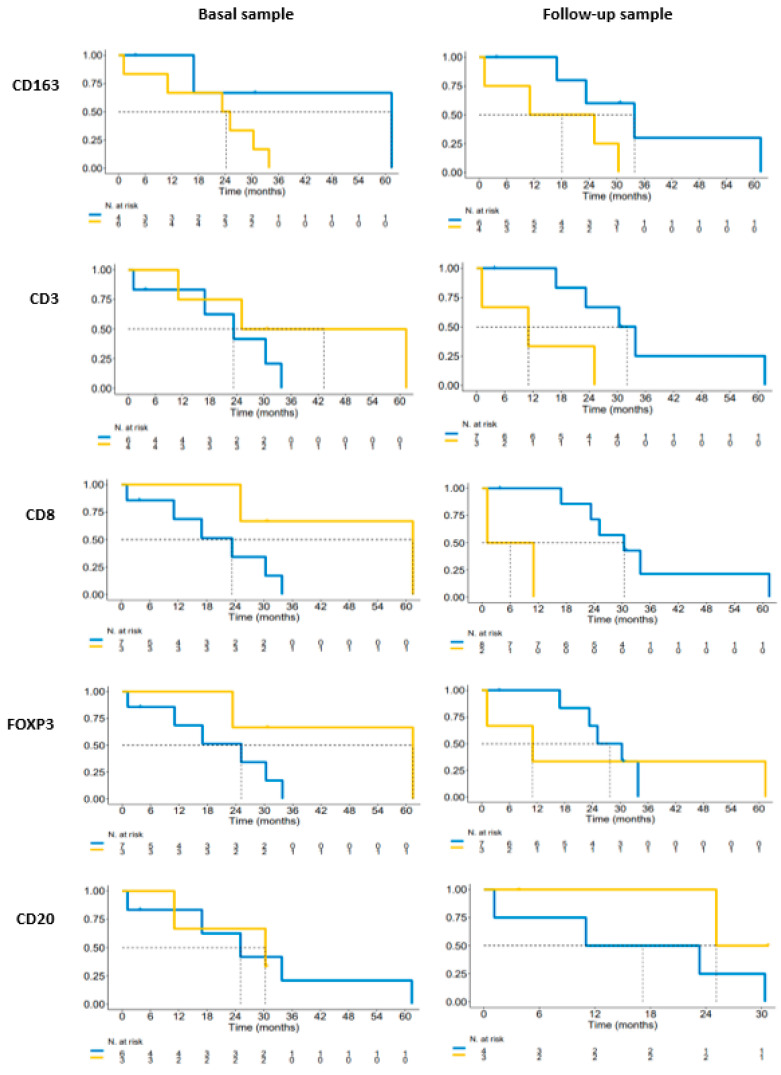
Kaplan–Meier overall survival according to TIL expression in matched samples. Blue lines represent levels of TILs below the median and yellow lines represents levels of TILs above the mean.

Median survival of the entire cohort was 25.1 m (16.9-NR). Analysis of OS in basal samples based on higher/lower density than the median for each marker was performed (Figure 5). According to the univariate analysis, in our series we found that in pretreatment samples, patients with a higher density of CD3, CD8, FOXP3, and CD20 and a lower density of CD163 presented a higher rate of survival. Median OS in pretreated samples for patients with a higher density of CD3 was 43.2 months vs. 23.3 months (*p* = 0.29), CD8 was 61.4 vs. 23.3 months (*p* = 0.13), FOXP3 was 61.4 months vs. 25.1 months (*p* = 0.16), and CD20 was 30.4 vs. 25.1 months (*p* = 1). Patients with a lower density of CD 163 in pretreatment samples presented a median survival of 61.4 months vs. 24.2 months (*p* = 0.17) compared to patients with a high density. The opposite results were found in the analysis of the second tumor sample, with a higher survival rate for patients with a lower density of CD3, CD8, FOXP3, and CD163 and a higher density of CD20. Median survival for patients in the second sample with a low density of CD3 was 32.1 vs. 11 months (*p* = 0.06), for patients with low CD8 it was 30.3 vs. 6.1 months (*p* = 1), for patients with low FOXP3 the median survival was 27.7 vs. 11 months (*p* = 0.87), and for patients with low CD163 the median survival was 33.8 vs. 18.1 months (*p* = 0.11). Patients with high CD20 presented a median survival of 25.1 months vs. 17.1 months for patients with lower levels (*p* = 0.21)

No relevant differences in overall response rate were detected based on the median of each TIL.

### 3.5. Gene Expression Analysis

**Figure 6:**
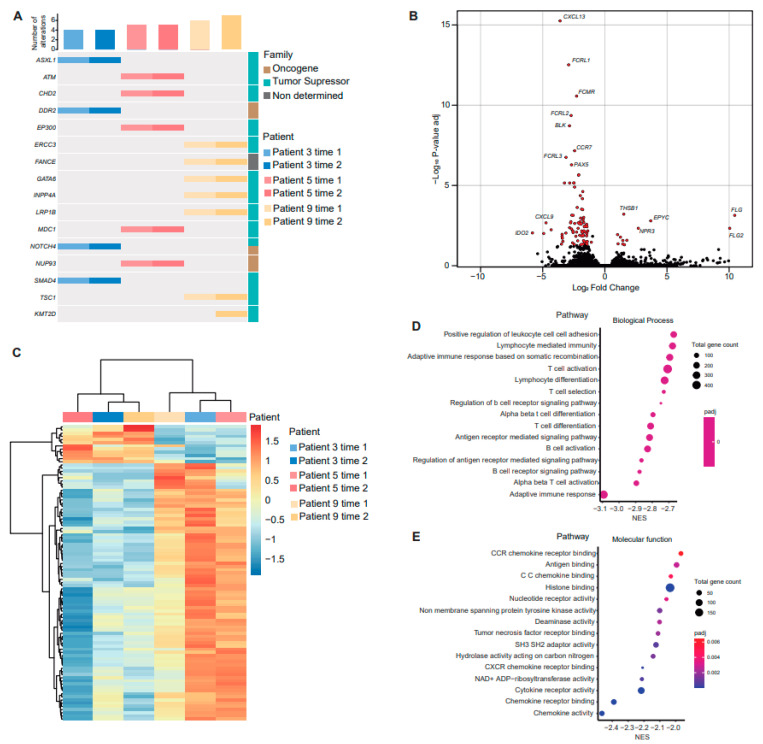
Analysis of gene expression in paired samples. (**A**) Oncoprint representation of an integrated annotation of somatic mutations for altered genes found in paired samples. Samples are arranged in columns with genes labelled along rows (number of alterations per sample on the top and gene classification on the right). (**B**) Volcano plot representing differences in gene expression between acquisition times. The *x*-axis indicates the fold-change (log-scaled); the *y*-axis indicates the *p*-values (log-scaled). Each symbol represents a different gene, and differentially expressed genes (DEG) are highlighted in red. *p* < 0.05 was considered to be statistically significant, and a fold change > 1 was set as the threshold value. (**C**) Heat map of the DEGs. The gradient from red to blue represents the change from down- to upregulation. Gene ontology enrichment analysis of downregulated DEGs showing biological process (**D**) and molecular function (**E**).

It was planned to perform genomic analysis in all of the paired samples, but only three patients had enough tumor material for DNA and RNA sequencing. Of these patients, two (patient 3 and patient 5) received the same systemic treatment (chemotherapy plus oncolytic virus), and the other patient (patient 9) received two lines of chemotherapy followed by anti PD-1. DNA-seq analysis did not show any relevant difference between paired samples, with almost the same mutations detected independently of acquisition time. Only a subclonal *KMT2D* appeared in the second biopsy of patient 9 (Figure 6A and Supplementary Table S3). As previously described, most mutations were identified in tumor suppressor genes, which have no therapeutic targets (Figure 6A)

The combined analysis of all the three patients with paired samples showed 93 significantly differentially expressed genes (DEG) between acquisition times (Figure 6B and Supplementary Tables S4 and S5). Between the most downregulated genes, we found *IDO2*, *CXCL9*, *IDO1*, *CXCL13*, *CXCL10*, *HLA-DOB*, *FCRL1*, and *FCRL2*. Cluster analysis of DEGs also confirmed that samples were mainly grouped by time of sample acquisition (Figure 6C). Of the 81 downregulated genes, 51 (63%) belonged to the “immune system process” (GO:002376), whereas of the 12 upregulated genes, 3 (25%) belonged to the “immune system process” (GO:002376). Finally, to explore which biological processes may be affected or be behind the transcriptomic changes, gene set enrichment analysis (GSEA) was performed based on the known gene ontology (GO) function of co-expressed genes (Figure 6D,E). The majority of the downregulated genes were related to adaptative immune response, including those affecting both B-cell and T-cell pathways. Our gene expression analysis suggests that the immune system tends to turn off after treatment or disease progression.

## 4. Discussion

In our study, we report the characteristics of TILs present in tumor samples of MPM patients and their evolution over time and after systemic treatment. We demonstrated, in our small series, that during the evolution of disease without any treatment there was an increase in the inflammatory component in tumor samples. After systemic treatment there was a decrease in the number of TILs and downregulation of immune-related genes.

The success of immunotherapy in melanoma and non-small cell lung cancer is thought to depend on pre-existing T-cell infiltration of tumors. The knowledge that TILs may be predictors of response to immunotherapy has led to increasing interest in the study of TILs, highlighting their role as a prognostic factor and predictor of chemotherapy or immunotherapy.

In MPM, CD4 and CD8 has been reported as the predominant TILs in untreated tumor samples [20]. Previous studies on MPM identified an association of TILs with histology. Pasello demonstrated in chemonaive samples that sarcomatoid and biphasic MPM were characterized by high CD8+ TILs, whereas epithelioid tumors presented higher CD4+ and CD20+ [31]. On the contrary, another study with 88 patients revealed that in sarcomatoid CD19+ and CD4+ were significantly enhanced [24]. In our series, we could not confirm the association of histology with the pattern of TILs because all but one patient had epithelioid tumors.

Several studies on MPM have confirmed that CD8+ is a prognosis marker in patients who have undergone surgery, but its prognosis remains unclear in chemotherapy-treated patients [20,21]. Contrary to what has been observed in patients treated with surgery, CD8+ was associated with poor prognosis in one series, but two larger series demonstrated that CD8+ conferred a survival advantage over chemotherapy [22,23,24]. Moreover, CD4+ TILs were associated with favorable survival in two studies [23,39]. In a study on MPM with paired samples, higher survival and disease-free survival were found for patients with low stromal scores and better survival was found for patients with high immune scores [40]. Regarding the prognostic role of TILs, the small number in our series did not allow for firm conclusions. However, we observed that high levels of CD3+, CD8+, and FOXP3 in pretreatment samples were positively correlated with improved survival.

Regarding the impact of TILs in response to chemotherapy, in a first series published by Marcq with 54 patients, 40 supplied untreated and 14 supplied treated not-matched samples, and it was demonstrated that samples from treated patients showed more infiltration [41]. Pasello performed an analysis in paired samples of 14 patients before and after chemotherapy with cisplatin plus pemetrexed, and after chemotherapy there was a significant increase in CD3+ T cells and a tendency for CD68+ macrophages and CD8+ to increase [31]. Dammeijer demonstrated that gemcitabine was significantly associated with an increase in NK cells [42]. In our series of 10 patients with paired samples, we included 3 patients without any treatment between samples and with disease progression among biopsies, and in this cohort, we found an increase in CD3+, CD8+, CD163+, and FOXP3 and a decrease in CD20+ over time. However, when we evaluated the seven pairs of samples of patients treated with chemotherapy, we found that after chemotherapy most of the patients presented a decrease in CD3+, CD8+, CD163+, CD20+, and FOXP3. Our results are contrary to those reported by Pasello et al. In their project, they detected an increase in CD3+ and CD8+ after chemotherapy, but we observed a decrease in CD3+ and CD8+, among others, after chemotherapy. Some possibilities that could explain these differences are the different antibodies used for the immune cell analysis, the lack of standardization for TIL analysis and TIL measures, or differences in the characteristics of patients who received more lines of treatment in our series. Additional prospective studies would provide more definitive conclusions. Our results suggests that over time there was an increase in the inflammatory infiltrate in MPM but that after chemotherapy the tumor turned colder. This observation could suggest that treatments with higher activity in inflamed tumors must be administered at an earlier stage of the disease.

The role of PD-L1 in response to treatment in MPM has been evaluated. In the series published by Marcq, PD-L1 was only detectable in untreated patients but not after patients treated with chemotherapy [41]. In a series of patients treated with nivolumab, the analysis of PD-L1 in a pre-treatment biopsy compared to on-treatment biopsy demonstrated that PD-L1 expression in neither pretreatment nor on-treatment biopsy specimens was correlated with outcome [36]. Finally, in a biomarker analysis of a study with atezolizumab plus bevacizumab, responses in both PD-L1+ and PD-L1–tumors were detected, median TMB was similar among responders and non-responders, and no differences between responders and non-responders in CD3+, CD8+, CD68+, and FOXP3 were found [43]. Two studies evaluated changes in the proportion of T cells in blood samples after treatment with immunotherapy and found that after tremelimumab there was an increase in CD8, nivolumab did not induce changes in the proportion of T cells, and the combination of nivolumab plus ipilimumab increased the proportion of CD4+ T cells [44,45]. In a phase 1 study evaluating biomarkers in paired samples following treatment with ONCOS 102 adenovirus, after the treatment an upregulation of PD-L1 and an infiltration of CD8+ T cells was found [46]. In our series, we found that PD-L1 expression after treatment in general did not change, except in one patient who was PD-L1 negative in baseline samples, and after two lines of chemotherapy PD-L1 was higher than 50%. These changes could be due to heterogeneity in the samples.

Large-scale genomic studies aiming at characterizing MPM have provided new insights into its classification, proposing molecular subdivision and a continuous model of mesothelioma with strong differences in the expression of proangiogenic and immune checkpoints [9,10]. A correlation between immune profile and the characterization of the tumor microenvironment has been published. In a study with 99 samples, gene expression analysis revealed 3 molecular subgroups representing different immune profiles. The group that comprised 40% of the samples was mainly represented by “immune deserts,” with little evidence of B-cell or T-cell infiltration, and it was enriched in the sarcomatoid and biphasic histologies [47]. Another immunoproteogenomic analysis of MPM tumor tissue from 12 treatment naïve MPM samples defined 2 disparate immunologic subtypes of MPM, 1 containing greater numbers of partially exhausted CD8+ T cells and the other containing more Tregs expressing high ICOS and CTLA4 [48]. In a study with a deconvolution approach in MPM, the histo-molecular gradients revealed two molecular subtypes of MPM associated with prognosis: The sarcomatoid was associated with infiltration of T cells and monocytes, fibroblasts, and endothelial cells, and the epithelioid component was preferentially associated with natural killer cells [49]. In MPM, there is consistent evidence that the mutational load is generally extremely low. Genomic studies have identified common recurring alterations in MPM, but none of them have been targetable. The MEDUSA project demonstrated that there are common key genomic events for clonal and subclonal evolution that lend themselves to focused drug development [50]. We performed a genomic analysis of paired samples only in three patients due to the lack of enough tumor tissue for the full analysis. We found in the three patients evaluated that after both systemic treatment and immunotherapy there was a downregulation of the immune system, especially in the pathways related to B-cell and T-cell activation. In our set of samples, tumors tended to turn off genes related to the immune system after treatment. However, more patients should be analyzed to confirm our observations in order to guide appropriate decisions about immunotherapy regimens.

There are several limitations to consider in this project. First, the number of patients was too small to draw significant conclusions. Our project was based on patients with paired tumor samples attending our institution. Moreover, we completed the genomic analysis only in three pairs of samples due to the small biopsies. Obtaining paired samples in patients with mesothelioma is not a routine procedure for the treatment. The only previous experience that has been reported in mesothelioma was the study performed by Pasello et al., who performed an analysis of samples obtained at diagnosis and after neoadjuvant treatment in patients who were candidates for surgery. Our patient population mainly included patients with more advanced stages who were not candidates for surgical treatment. The results of our series should be validated in prospective studies with larger number of patients. Second, spatial and intratumoral heterogeneity has been demonstrated in MPM, suggesting that a single tumor biopsy specimen may be insufficient to fully characterize the tumor [50,51]. Additionally, the long period of time between some pairs of samples could influence the reactivity of antigens. Third, serial analysis of peripheral blood mononuclear cells (PBMC) has demonstrated dynamic changes of TILs within treatment. In our series, we had patients with paired samples after treatment, but the population of TILs in the second tumor biopsy was not representative of the real changes in TILs over time. In future research, it would be interesting to investigate the dynamics of intrapatient TILs in MPM.

## 5. Conclusions

In conclusion, our integrated analysis of paired samples with immune profile and genomic changes in MPM demonstrates that during the evolution of the disease the immune system tends to turn off. Immune checkpoint inhibitors are changing the landscape of treatment for patients with solid tumors, including MPM. However, not all patients show favorable response. Therefore, it is crucial to find predictive biomarkers that enable us to withhold treatment from patients who are unlikely to respond. Larger validation cohorts are needed to determine the best treatment schedule considering dynamic changes to the immune profile.

## Figures and Tables

**Table 1:** Antibodies used for immunohistochemical analysis.

Antibody	Company	Reference	Antigen Retrieval	Primary Ab	Detection Kit	Detection Kit Incubation Time	Chromogen	Counterstaining
PD-L1	ROCHE	741–4905	Ultra CC1 64 min 100 °C	16 min 36 °C RTU	OV OptiView DAB	OV HQ Universal linker 8 min OV HRP Multimer 8 min	OV DAB + OV H202	Hematoxylin II 8 min + bluing reagent 4 min
CD20	ROCHE	760–2531	Ultra CC1 36 min 95 °C	32 min RT RTU	UV UltraView DAB	UV HRP UNIV MULT 8 min	UV DAB + UV DAB H_2_O_2_ 8 min	Hematoxylin II 4 min + bluing reagent 4 min

**Table 2:** Antibodies used for next-generation immunohistochemical analysis.

Antibody	Company	Reference	Antigen Retrieval	Primary Antibody	Secondary Antibody	Chromogen
FOXP3	ABCAM	AB99963	CC1 92′ 95°	1 h	HRP RB 20′	AECPLUS. 150UL
CD8/144B	DAKO	M7103	CC2 8′ 100° CC1 40′ 95°	32′ 37° 1/100	HRP MS 8′	AECPLUS. 200UL
CD3(2GV6)	ROCHE	790–4341	CC2 8′ 100° CC1 40′ 95°	40′ 36°	HRP RB 8′	AECPLUS. 200UL
CD163	ROCHE	760–4437	CC2 8′ 100° CC1 64′ 95°	48′ 37°	HRP MS 8′	AECPLUS. 200UL
KI67	ROCHE	790–4286	CC2 8′ CC1 64′ 95°	52′ 37°	HRP RB 8′	AECPLUS. 200UL
PanCK (AE1/AE3)	PALEX	PDM072	CC2 8′ 100° CC1 40′ 95°	24′ 36°	HRP MS 8′	AECPLUS. 200UL

**Table 3:** Clinical characteristics of patients.

	Gender	Age	Histology	Clinical Stage	Asbestos Exposure	Time between Biopsies (Months)	Treatment between Biopsies	OS (Months)
Patient 1	F	53	Epithelioid	II	Yes	7	None	25
Patient 2	F	54	Epithelioid	II	Yes	10	Cisplatin–pemetrexed Anetumab–ravtansine	21
Patient 3	M	71	Epithelioid	II	Yes	10	Cisplatin–pemetrexed Oncolytic virus	39
Patient 4	M	62	Epithelioid	III	Yes	10	Cisplatin–pemetrexed	23
Patient 5	M	60	Epithelioid	III	Yes	14	Cisplatin–pemetrexed Oncolytic virus	31
Patient 6	F	83	Epithelioid	III	Yes	8	None	17
Patient 7	M	84	Biphasic	III	Yes	1	None	19
Patient 8	M	62	Epithelioid	II	No	3	Cisplatin–pemetrexed	43
Patient 9	M	69	Epithelioid	III	Yes	33	Cisplatin–pemetrexed–bevacizumab, pembrolizumab	34
Patient 10	M	59	Epithelioid	III	Yes	16	Cisplatin–pemetrexed, vinorelbine	17

F: female, M: male.

## Data Availability

The cohort-level data presented in this study are available in this article; however, individual values per patient are available on request from the corresponding author.

## Supplementary Materials

The following supporting information can be downloaded at: https://www.mdpi.com/article/10.3390/cancers15143611/s1, Table S1. NGS panel; Table S2. patients characteristics, Table S3. DNA analysis; Table S4. Down-regulated genes; Table S5. Up-regulated genes.
