# Supplementary material for: Expression of TILs and Patterns of Gene Expression from Paired Samples of Malignant Pleural Mesothelioma (MPM) Patients
Source: Cancers (Basel). 2023 Jul 14;15(14):3611. doi: 10.3390/cancers15143611 (PMC10377125; doi:10.3390/cancers15143611)
Supplement: Supplementary file 1 [file cancers-15-03611-s001.zip › cancers-2314858-supplementary/Supplementary Table S1. NGS panel.pdf]

Supplemental Table 1. VHIO-300. List of genes included in the targeted capture panel.

ABL1	CBFB	EIF1AX	FOXL2	IRS2	MSH6	PIK3R1	ROS1	TGFBR2
ABL2	CBL	EP300	FOXP1	JAK1	MTOR	PIK3R2	RPS6KA4	TMEM127
ACVR1B	CCND1	EPCAM	FRS2	JAK2	MUTYH	PIK3R3	RPS6KB2	TMPRSS2
AKT1	CCND2	EPHA3	FUBP1	JAK3	MYC	PIM1	RPTOR	TNFAIP3
AKT2	CCND3	EPHA5	GABRA6	JUN	MYCL	PLCG2	RSPO2	TNFRSF14
AKT3	CCNE1	EPHA7	GATA1	KAT6A	MYCL1	PLK2	RUNX1	TOP1
ALK	CD274	EPHB1	GATA2	KDM5A	MYCN	PMAIP1	RUNX1T1	TOP2A
ALOX12B	CD276	ERBB2	GATA3	KDM5C	MYD88	PMS1	RYBP	TP53
AMER1	CD79A	ERBB3	GATA4	KDM6A	MYOD1	PMS2	SDHA	TP63
APC	CD79B	ERBB4	GATA6	KDR	NBN	PNRC1	SDHAF2	TRAF7
AR	CDC73	ERCC2	GID4	KEAP1	NCOR1	POLD1	SDHB	TSC1
ARAF	CDH1	ERCC3	GLI1	KEL	NF1	POLE	SDHC	TSC2
ARFRP1	CDK12	ERCC4	GNA11	KIT	NF2	PPP2R1A	SDHD	TSHR
ARID1A	CDK4	ERCC5	GNA13	KLF4	NFE2L2	PPP2R2A	SETD2	U2AF1
ARID1B	CDK6	ERG	GNAQ	KLHL6	NFKBIA	PRDM1	SF3B1	VEGFA
ARID2	CDK8	ERRFI1	GNAS	KMT2A	NKX2-1	PREX2	SH2D1A	VHL
ARID5B	CDKN1A	ESR1	GPR124	KMT2C	NKX3-1	PRKAR1A	SHH	VTCN1
ASXL1	CDKN1B	ETV1	GREM1	KMT2D	NOTCH1	PRKCI	SHQ1	WISP3
ASXL2	CDKN2A	ETV6	GRIN2A	KRAS	NOTCH2	PRKDC	SLIT2	WT1
ATM	CDKN2B	EZH2	GRM3	LATS1	NOTCH3	PRSS8	SMAD2	XIAP
ATR	CDKN2C	FAM175A	GSK3B	LATS2	NOTCH4	PTCH1	SMAD3	XPO1
ATRX	CEBPA	FAM46C	H3F3A	LMO1	NPM1	PTEN	SMAD4	YAP1
AURKA	CHD2	FANCA	H3F3C	LRP1B	NRAS	PTPN11	SMARCA4	YES1
AURKB	CHD4	FANCC	HGF	LYN	NSD1	PTPRD	SMARCB1	ZBTB2
AXIN1	CHEK1	FANCD2	HIST1H1C	LZTR1	NTRK1	PTPRS	SMARCD1	ZNF217
AXIN2	CHEK2	FANCE	HIST1H2BD	MAD2L2	NTRK2	PTPRT	SMO	ZNF703
AXL	CIC	FANCF	HIST1H3B	MAGI2	NTRK3	QKI	SNCAIP	
B2M	CREBBP	FANCG	HNF1A	MALT1	NUP93	RAC1	SOCS1	
BAP1	CRKL	FANCL	HRAS	MAP2K1	PAK1	RAD50	SOX10	
BARD1	CRLF2	FANCM	HSD3B1	MAP2K2	PAK3	RAD51	SOX17	
BBC3	CSF1R	FAS	HSP90AA1	MAP2K4	PAK7	RAD51B	SOX2	
BCL2	CTCF	FAT1	ICOSLG	MAP3K1	PALB2	RAD51C	SOX9	
BCL2L1	CTLA4	FBXW7	IDH1	MAP3K13	PARK2	RAD51D	SPEN	
BCL2L11	CTNNA1	FGF10	IDH2	MAP3K5	PARP1	RAD52	SPOP	
BCL2L2	CTNNB1	FGF14	IFNGR1	MAPK1	PAX5	RAD54L	SPTA1	
BCL6	CUL3	FGF19	IGF1	MAPK7	PBRM1	RAF1	SRC	
BCOR	CYLD	FGF23	IGF1R	MAX	PDCD1	RANBP2	STAG2	
BCORL1	DAXX	FGF3	IGF2	MCL1	PDCD1LG2	RARA	STAT3	
BLM	DCUN1D1	FGF4	IKBKE	MDC1	PDGFRA	RASA1	STAT4	
BMPR1A	DDR2	FGF6	IKZF1	MDM2	PDGFRB	RB1	STK11	
BRAF	DICER1	FGFR1	IL10	MDM4	PDK1	RBM10	STK40	
BRCA1	DIS3	FGFR2	IL7R	MED12	PDPK1	RECQL4	SUFU	
BRCA2	DNMT1	FGFR3	INHBA	MEF2B	PHOX2B	REL	SUZ12	
BRD4	DNMT3A	FGFR4	INPP4A	MEN1	PIK3C2B	RET	SYK	
BRIP1	DNMT3B	FH	INPP4B	MET	PIK3C2G	RFWD2	TAF1	
BTG1	DOT1L	FLCN	INSR	MITF	PIK3C3	RHEB	TBX3	
BTK	E2F3	FLT1	IRAK4	MLH1	PIK3CA	RHOA	TERT	
C11ORF30	EED	FLT3	IRF2	MPL	PIK3CB	RICTOR	TET1	
CARD11	EGFL7	FLT4	IRF4	MRE11A	PIK3CD	RIT1	TET2	
CASP8	EGFR	FOXA1	IRS1	MSH2	PIK3CG	RNF43	TGFBR1	
