# Supplementary material for: Expression of TILs and Patterns of Gene Expression from Paired Samples of Malignant Pleural Mesothelioma (MPM) Patients
Source: Cancers (Basel). 2023 Jul 14;15(14):3611. doi: 10.3390/cancers15143611 (PMC10377125; doi:10.3390/cancers15143611)
Supplement: Supplementary file 1 [file cancers-15-03611-s001.zip › cancers-2314858-supplementary/Supplementary Table S2 clinical characteristics.pdf]

Supplementary Table 2: systemic treatments for each patient

**Nº patient**	**1st line**	**ORR**	**PFI (m)**	**2nd line**	**ORR**	**PFI (m)**	**3rd line**	**ORR**	**PFI (m)**	**4rd line**
1	None									
2	CDDP-pem	PR	6	*Anetumab	PR	8				
3	CDDP-pem-Virus	PR	5	*Carbo-pem	SD	6	Nivo+ipi	SD	4m	
4	CDDP-pem	PR	10	*CDDP-pem	SD	4				
5	CDDP-pem-Virus	SD	12	*CDDP-pem	PR	5	antiPD1	PD		
6	None									
7	None									
8	CDDP-pem	SD	NR							
9	CDDP-pem-beva	PR	8	Carbo-pem	PD	0	Pembro	PR	6m	*LAG3
10	CDDP-pem	PD		Vinorelbine	PR	10	*Gemcitabine	PD		

(*) means the time of second biopsy (performed before to initiate the therapy). Pem: pemetrexed; beva: bevacizumab; ORR: overall response rate, PR: partial response, SD: stable disease, PD: progressive disease, PFI: progression free interval, m: months, NR: not reached
